# Heterogeneity in recombination rates and accessory gene co-occurrence distinguish *Pseudomonas aeruginosa* phylogroups

**DOI:** 10.1128/msystems.00301-25

**Published:** 2025-04-30

**Authors:** Samara T. Choudhury, Kathryn R. Piper, Manuela Montoya-Giraldo, Odion O. Ikhimiukor, Jeremy R. Dettman, Rees Kassen, Cheryl P. Andam

**Affiliations:** 1 Department of Biological Sciences, University at Albany, State University of New York 1085, Albany, New York, USA; 2 Ottawa Research and Development Centre, Agriculture & Agri-Food Canada 98674 , Ottawa, Ontario, Canada; 3 Department of Biology, McGill University 98614, Montreal, Québec, Canada; University of California, Los Angeles, Los Angeles, California, USA

**Keywords:** *Pseudomonas aeruginosa*, core genome, accessory genome, phylogroup, recombination

## Abstract

*Pseudomonas aeruginosa* (class Gammaproteobacteria) is a ubiquitous, ecologically widespread, and metabolically versatile species. It is also an opportunistic pathogen that causes a variety of chronic and acute infections in humans. Its ability to thrive in diverse environments and exhibit a wide range of phenotypes lies in part on its large gene pool, but the processes that govern inter-strain genomic variation remain unclear. Here, we aim to characterize the recombination features and accessory genome structure of *P. aeruginosa* using 840 globally distributed genome sequences. The species can be subdivided into five phylogenetic sequence clusters (corresponding to known phylogroups), two of which are most prominent. Notable epidemic clones are found in the two phylogroups: ST17, ST111, ST146, ST274, and ST395 in phylogroup 1, and ST235 and ST253 in phylogroup 2. The two phylogroups differ in the frequency and characteristics of homologous recombination in their core genomes, including the specific genes that most frequently recombine and the impact of recombination on sequence diversity. Each phylogroup’s accessory genome is characterized by a unique gene pool, co-occurrence networks of shared genes, and anti-phage defense systems. Different pools of antimicrobial resistance and virulence genes exist in the two phylogroups and display dissimilar patterns of co-occurrence. Altogether, our results indicate that each phylogroup displays distinct histories and patterns of acquiring exogenous DNA, which may contribute in part to their predominance in the global population. Our study has important implications for understanding the genome dynamics, within-species heterogeneity, and clinically relevant traits of *P. aeruginosa*.

**IMPORTANCE:**

The consummate opportunist *Pseudomonas aeruginosa* inhabits many nosocomial and non-clinical environments, posing a major health burden worldwide. Our study reveals phylogroup-specific differences in recombination features and co-occurrence networks of accessory genes within the species. This genomic variation partly explains its remarkable ability to exhibit diverse ecological and phenotypic traits, and thus contribute to circumventing clinical and public health intervention strategies to contain it. Our results may help inform efforts to control and prevent *P. aeruginosa* diseases, including managing transmission, therapeutic efforts, and pathogen circulation in non-clinical environmental reservoirs.

## INTRODUCTION

The Gram-negative *Pseudomonas aeruginosa* (class Gammaproteobacteria) is a ubiquitous and metabolically versatile microbe that inhabits many disparate ecological niches, from aquatic and soil habitats to animals and plants (1–3). *P. aeruginosa* is also an opportunistic human pathogen associated with many life-threatening acute and chronic infections (4). These include bronchiectasis, ventilator-associated pneumonia, chronic obstructive pulmonary disease, urinary tract infection, otitis externa, burn and wound injuries, bone and joint infections, bacteremia, and systemic infections (4). It is the most common chronic colonizer of the respiratory tracts of adult patients with cystic fibrosis (CF), causing a persistent inflammatory response and subsequently a gradual decline of lung function (5). Approximately 60%–70% of adult CF patients suffer from persistent *P. aeruginosa* infections, and this leads to a higher risk of mortality and morbidity (6, 7). *P. aeruginosa* is one of the World Health Organization’s six ESKAPE pathogens, which refer to a group of bacterial species that are a major cause of nosocomial infections worldwide, are multidrug resistant, and are priority targets for the development of novel antimicrobials (8). From 1990 to 2021, an estimated 9.4% of global deaths of persons ≥5 years and 8.6% of persons <5 years are attributable to antimicrobial-resistant *P. aeruginosa* (9). Of major concern is carbapenem-resistant *P. aeruginosa* that ranks fourth in terms of attributable burden globally (9).

*P. aeruginosa*’s ability to thrive in a variety of environments, whether free-living or as harmless colonizer or consummate pathogen, lies in part on its large genetic repertoire (10–13). Its accessory genome, i*.*e., consisting of genes present in one or few strains (14), is remarkably large, with numerous transporters, catabolic genes, transcriptional regulators, and two-component regulatory systems (10, 11). The seemingly limitless supply of these adaptive genes manifests itself in *P. aeruginosa*’s resilience to stress, even under repeated exposure to antimicrobials (15, 16). Moreover, a suite of mobile genetic elements such as phages, plasmids, integrons, transposons, and integrative and conjugative elements is prevalent in *P. aeruginosa*, facilitating the rapid horizontal acquisition of accessory genes (10, 17, 18). Inter-strain differences in phenotypic attributes such as antimicrobial susceptibility (19), epidemic potential (20, 21), virulence-related features (e*.*g., pyocyanin production, biofilm formation, twitching motility) (20, 22), and adaptive trajectories in response to growth media (23, 24) have also been documented. Such remarkable variation poses a challenge in developing effective therapeutic interventions, which is a significant barrier to treating *P. aeruginosa* infections.

Here, we aim to characterize the recombination features and accessory genome structure of *P. aeruginosa* lineages. We analyzed 840 genome sequences derived from 35 countries in six continents, spanning the years from 1936 to 2014, and from different ecological sources. Two dominant phylogenetic lineages (or phylogroups), each comprising multiple sequence types (STs), differ in the frequency and characteristics of homologous recombination in their core genomes, accessory gene co-occurrence, and defense systems against exogenous DNA. Our findings will help inform efforts to control and prevent *P. aeruginosa* diseases, including managing transmission, therapeutic strategies, and circulation in non-clinical environmental reservoirs.

## RESULTS

### Two dominant phylogroups dominate the global population of *P. aeruginosa*

**Fig 1:**
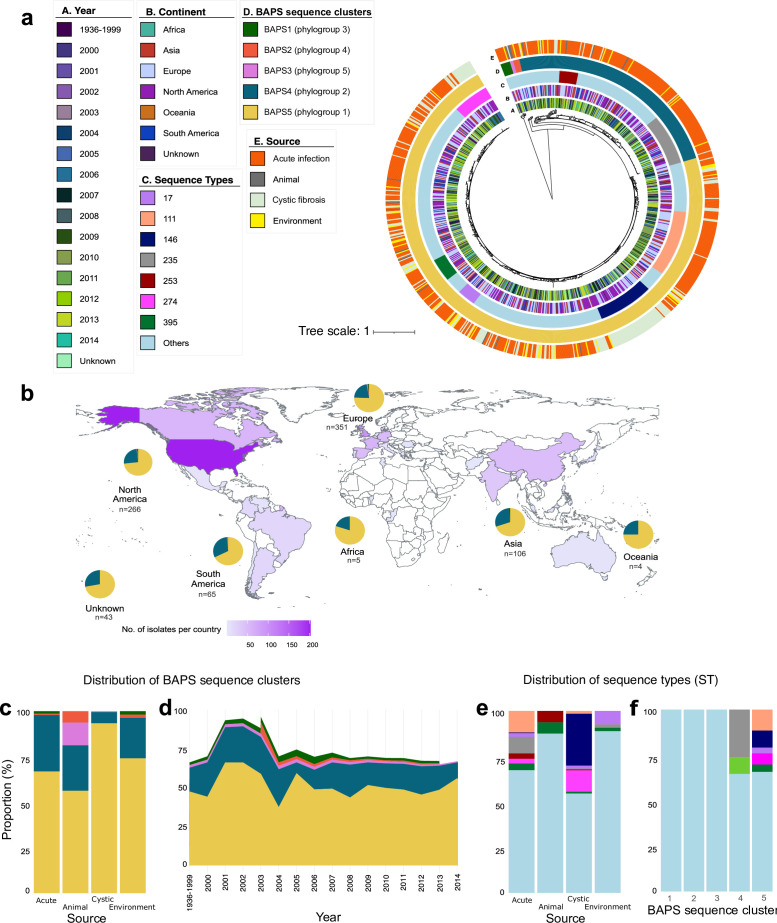
Population structure and phylogenetic relationship of 840 *P*. *aeruginosa* genomes. (a) Midpoint-rooted maximum likelihood tree constructed with SNPs from concatenated 4,088 core genes and 1,037 soft core genes. The tree scale represents the number of nucleotide substitutions per site. The five outer rings depict the year of sampling, geographical origin (continent), ST, BAPS sequence clusters (corresponding to phylogroups), and ecological source. For visual clarity, only the seven major STs are displayed. (b) Global map displaying the regions from which the *P. aeruginosa* strains were isolated. The pie charts illustrate the proportion of the five BAPS sequence clusters in each major geographical region or continent. The number of genomes derived from each region is displayed below each pie chart. Proportion of BAPS sequence clusters according to ecological source (c) and per year (d). Proportion of STs according to ecological source of isolates (e) and BAPS sequence clusters (f).

We retrieved 840 publicly available genome sequences of *P. aeruginosa* compiled and previously published in reference 12 (Fig. 1a; Table S1). The sequences were derived from 35 countries across six continents, spanning the years from 1936 to 2014. They came from four ecological sources: cystic fibrosis (*n* = 196 genomes), acute infections (*n* = 574), non-clinical environment (*n* = 54), and animals (*n* = 16). The totality of gene families across the entire data set, or pan-genome (25), consisted of 28,019 genes, which can be categorized into 4,088 core genes (present in >99% of genomes), 1,037 soft core genes (present in 95% to <99% of genomes), 1,573 shell genes (present in 15% to <95% of genomes), and 21,321 cloud genes (present in <15% of genomes) (Table S2). Our calculation of the core genes is comparable to previously reported estimates of approximately 5,000 genes (26–28), although other studies have determined a much smaller number (13, 29).

Population structure analysis using Bayesian hierarchical clustering of the core genome alignment showed five distinct sequence clusters, which correspond to phylogenetic lineages or phylogroups (Fig. 1a). We refer to these as phylogroups 1–5 following the nomenclature in previous pan-genome analyses of *P. aeruginosa* (12, 13). Two phylogroups are predominant in our data set (phylogroup 1 [sequence cluster BAPS5] and phylogroup 2 [BAPS4]), consisting of 616 (73.33% of the total population) and 206 (24.52% of the total population) genomes, respectively. Phylogroups 3–5 each contained less than 10 genomes. Phylogroups 1 and 2 were found in six continents (Fig. 1b), four ecological sources (Fig. 1c), and all years of sampling (Fig. 1d).

Using *in silico* multi-locus sequence typing (MLST), which partitions the population based on allelic variation in seven housekeeping genes and assigns an ST to strains (30), we detected the presence of 261 previously identified STs. Seven STs (STs 17, 111, 146, 235, 253, 274, and 395) each consisted of >15 genomes, and all together represented 33.81% of the data set. The distribution of the seven STs varied by ecological source (Fig. 1e) but was mainly found in the two major phylogroups: ST17, ST111, ST146, ST274, and ST395 in phylogroup 1 (BAPS5), and ST235 and ST253 in phylogroup 2 (BAPS4) (Fig. 1f). ST146 and ST274 genomes were derived mainly from cystic fibrosis cases, while the other five major STs predominated in acute infections with the other ecological sources intermingled among them. However, given the low numbers of genomes per ST, these results should only be interpreted within the confines of this data set.

### Frequent but variable features of homologous recombination in the core genome

**Fig 2:**
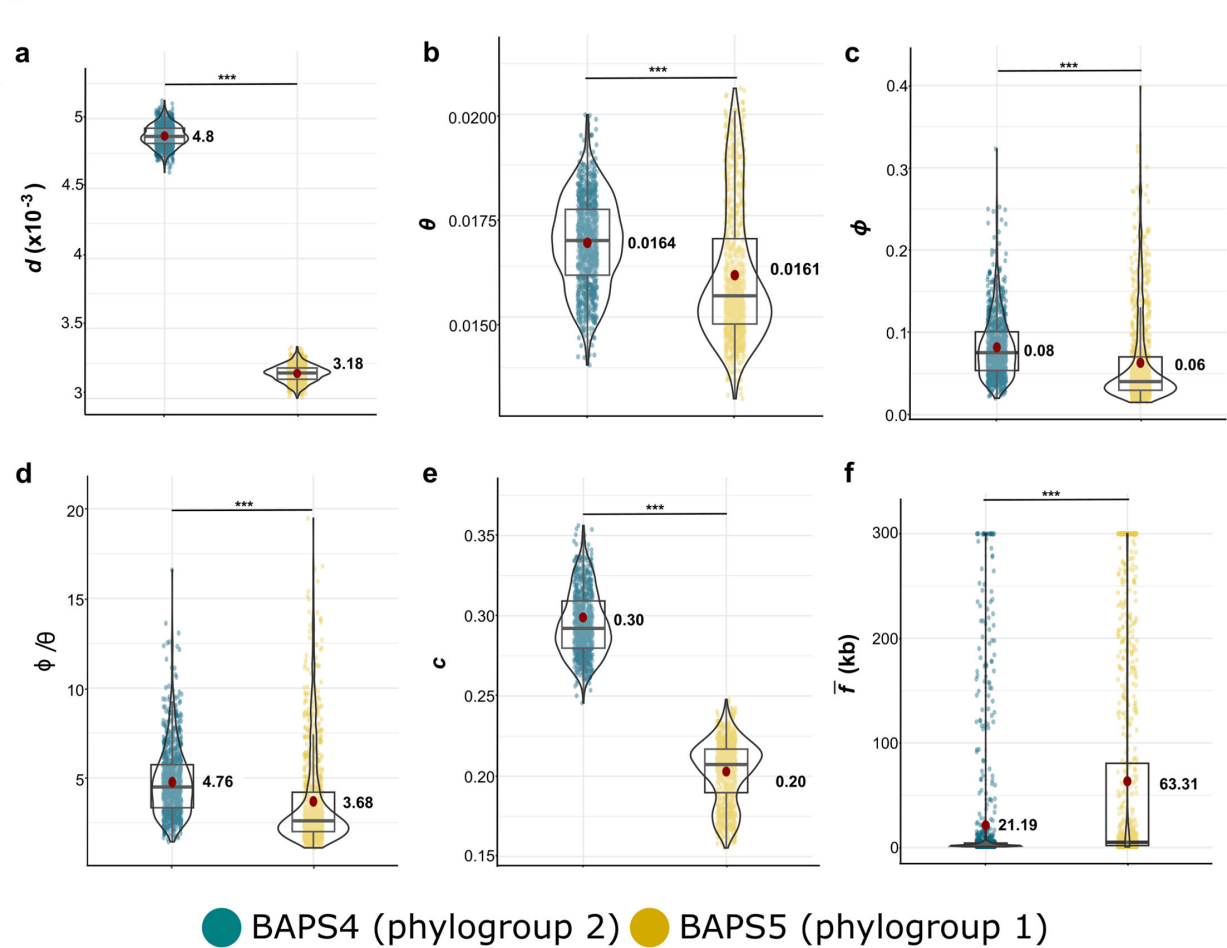
Recombination parameters calculated using Mcorr in sequence clusters BAPS4 (phylogroup 2) and BAPS5 (phylogroup 1). The core genome alignment of each sequence cluster was used as input in Mcorr. (a) *d*, diversity brought into the population by recombination and mutation; (b) θ, mutational divergence; (c) ϕ, recombinational divergence; (d) γ/μ (or ϕ/θ), relative rate of recombination to mutation; (e) *c*, recombination coverage; (f) *f̅* , mean fragment size of a recombination event. Violin jitter plots show the 1,000 bootstrap replicates. The red dots represent the mean. Box plots show the 25th, 50th, and 75th percentiles. Welch’s *t*-test was used for all comparisons; n.s., not significant; ***P < 0.0001. Details of Mcorr results are found in Table S3.

We next sought to determine the extent to which recombination has affected the genome evolution of phylogroups 1 (BAPS5) and 2 (BAPS4). We focus on these two phylogroups because they consist of a sufficient number of genomes to carry out recombination detection. Using the core genome alignment as input and 1,000 bootstrapped replicates, we used Mcorr to calculate six recombination parameters (31) (Fig. 2a through f; Table S3; Fig. S1). Here, Mcorr measures the recombination on each branch of the phylogeny. The diversity (*d*) refers to diversity stemming from both recombination and mutation, and was estimated to be 4.8 × 10^−3^ and 3.18 × 10^−3^ in phylogroup 2 and phylogroup 1, respectively (*P* value =  2.35 × 10^−11^; Welch’s *t*-test). The mutational divergence (θ), which refers to the mean number of mutations per locus since the divergence of a pair of homologous nucleotide sites in the sequence alignment, was estimated to be 0.0164 and 0.0161 in phylogroup 2 and phylogroup 1, respectively (*P* value =  5.82 × 10^−12^; Welch’s *t*-test). Recombinational divergence (ϕ) is the mean number of recombinations per locus since the divergence of a pair of homologous nucleotide sites in the sequence alignment and was estimated to be 0.08 and 0.06 in phylogroup 2 and phylogroup 1, respectively (*P* value =  6.66 × 10^−12^; Welch’s *t*-test). The ratio ϕ/θ gives the relative rate of recombination to mutation and was estimated to be 4.76 and 3.76 in phylogroup 2 and phylogroup 1, respectively (*P* value =  1.05 × 10^−11^; Welch’s *t*-test). We calculated the recombination coverage (*c*), which represents the fraction of the sequence alignment that harbors genetic diversity stemming from recombination events (*c* = 0 indicates clonal evolution; *c* = 1 indicates complete recombination). We estimated c to be 0.30 and 0.20 in phylogroup 2 and phylogroup 1, respectively (*P* value =  4.51 × 10^−11^; Welch’s *t*-test), indicating that 30% and 20% of sites in any one genome from phylogroup 2 and phylogroup 1, respectively, have experienced recombination. Lastly, the median fragment size (*f̅*) of a recombination event was estimated to be 2.06 kbp with a range of 758.50 bp to 299 kb for phylogroup 2 (mean = 21.19 kbp) and 5.03 kbp with a range of 667 bp to 300 kb for phylogroup 1 (mean = 63.31 kbp) (*P* value = <0.001; Welch’s *t*-test). For context, our results are comparable to those calculated for 281 genomes of a clinical *P. aeruginosa* population (*d* [0.014], θ [0.027], ϕ [0.29]), but have higher ϕ/θ (11), *c* (52%), and *f̅* (590 bp) (31). Overall, our data set shows that while the number of recombination events is relatively low, these recombination events appear to have had a large impact on the genome sequences. Our results are consistent with the outcomes of a previous study that showed recombination variation between lineages of *P. aeruginosa* using a maximum likelihood inference of recombination (32).

**Fig 3:**
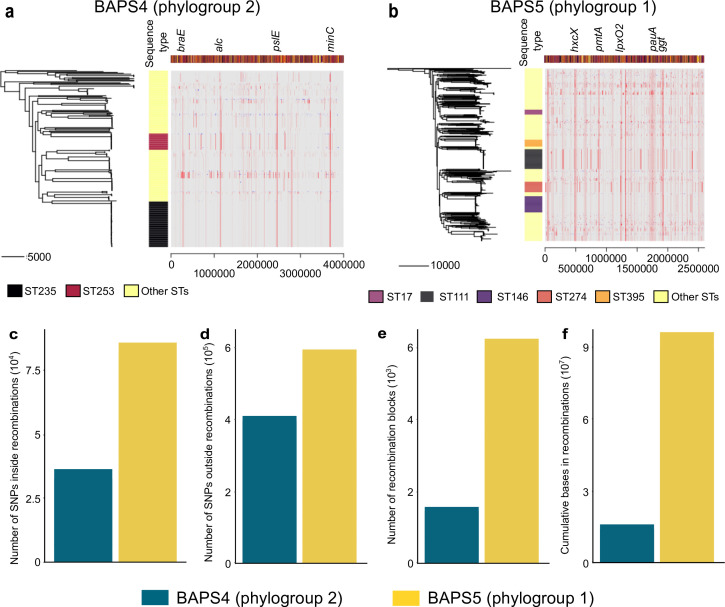
Recombination events calculated using Gubbins in sequence clusters BAPS4 (phylogroup 2) and BAPS5 (phylogroup 1). A core genome phylogeny was built for each sequence cluster (a) BAPS4 and (b) BAPS5. STs are shown as colored blocks, with only the most common STs shown for visual clarity. The matrix shows the inferred recombination events along the length of each sequence alignment. Each row in the matrix corresponds to a genome in the phylogeny, and each column represents a gene annotated using the reference strain PAO1 (accession no. SAMN02603714). Gene annotations of the reference genome are presented in different colored blocks across the length of the reference genome shown above the matrix. Within the matrix are red blocks representing recombination shared by multiple genomes, and blue blocks representing recombination events detected in a single genome. For visual clarity, only the most frequently recombined genes are labeled. Comparison of the (c) number of SNPs inside recombination blocks, (d) number of SNPs outside recombination blocks, (e) number of recombination blocks, and (f) cumulative bases in recombination blocks. Details of Gubbins results are found in Table S4.

We further sought to identify specific core genes in each of the two major phylogroups that have experienced recombination. Using a scanning window approach implemented in Gubbins (33), we identified regions of elevated density of single nucleotide polymorphisms (SNPs) across the core genome alignment, which Gubbins infers to have been generated by recombination (Fig. 3a and b). Results show a distinct set of recombined DNA segments throughout the length of each genome in each phylogroup, with phylogroup 2 (BAPS4) genomes experiencing more recombination events than phylogroup 1 genomes (Fig. 3; Table S4). In phylogroup 2, frequently recombining genes have functions related to amino acid transport (*braE*)*,* purine degradation (*alc*)*,* exopolysaccharide synthesis (*pslE*), and chromosomal segregation and division (*minC*) (Fig. 3a). In phylogroup 1 (BAPS5) genomes, we detected frequent recombination in *minC* as well as in genes associated with type II secretion system (*hxcX*)*,* response to oxidative stress and phagocytosis, pyocyanin expression and biofilm formation (*pmtA*)*,* lipopolysaccharide biosynthesis (*lpxO2*)*,* polyamine utilization (*pauA*), and glutathione degradation (*ggt*) (Fig. 3b). While the set of recombined genes differed between the two phylogroups, many of them are known to be associated with pathogenesis (4, 5).

Furthermore, we calculated four recombination metrics for every genome in each phylogroup using Gubbins: number of SNPs inside inferred recombination regions (Fig. 3c); number of SNPs outside inferred recombination regions (Fig. 3d); number of recombination blocks (Fig. 3e); and cumulative bases in recombinations, which refer to the total number of nucleotide bases in the alignment affected by recombination on a branch and its ancestors (Fig. 3f). The four metrics show that the frequency and impact of recombination were greater in phylogroup 1 (BAPS5) than in phylogroup 2 (BAPS4). We observed a difference in the propensity for different phylogroups to accumulate SNPs: phylogroup 1 (BAPS5) has consistently many SNPs both within and outside of recombination blocks, whereas phylogroup 2 (BAPS4) not only has fewer SNPs on average but also fewer within recombination blocks than outside of them.

### Relationship between recombination and defense systems

**Fig 4:**
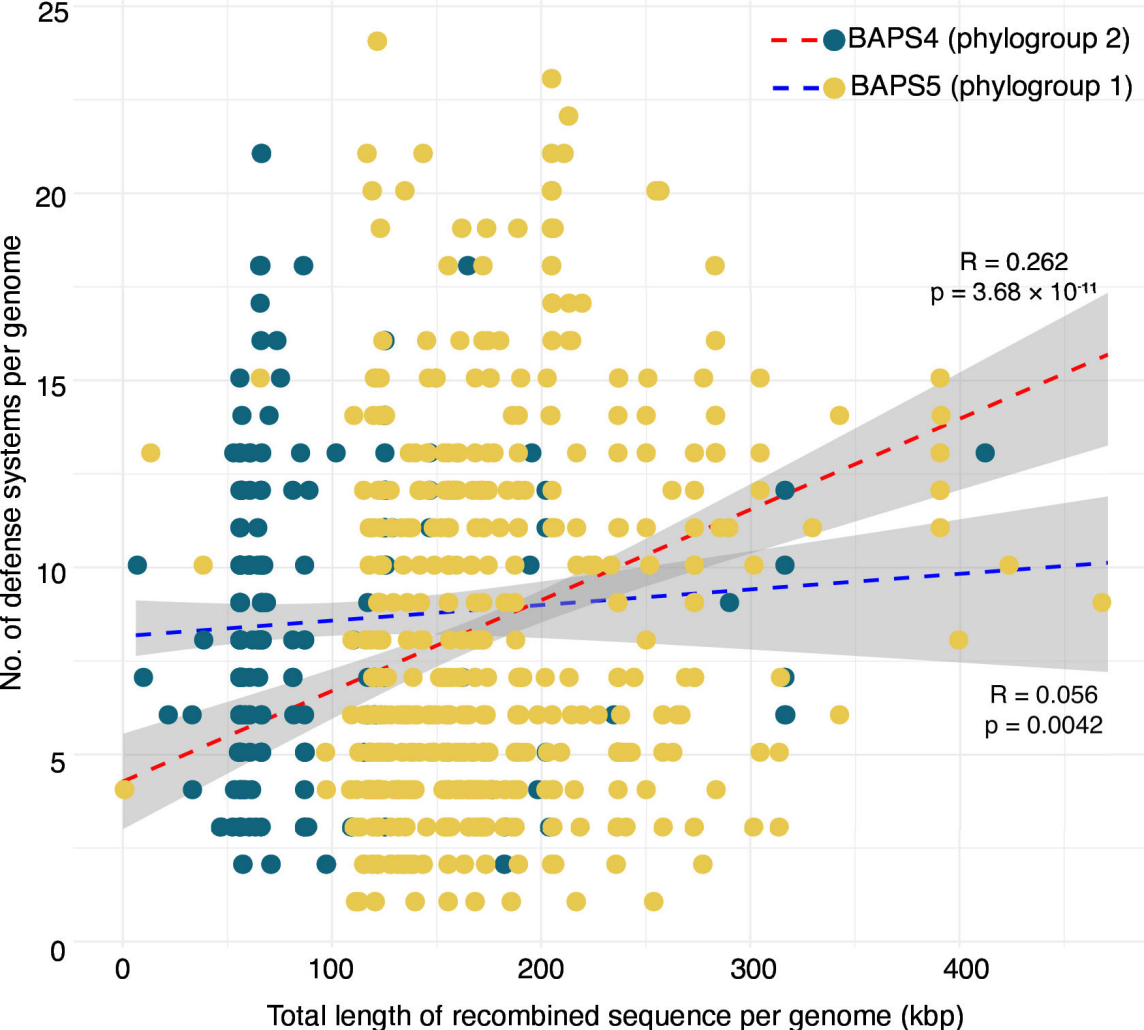
Relationship between recombination and defense systems in sequence clusters BAPS4 (phylogroup 2) and BAPS5 (phylogroup 1). Defense systems against exogenous and phage DNA were identified using DefenseFinder. Linear regression model of the total length of recombined sequence per genome (kbp) and the number of defense systems per genome. Each point represents a genome, with colors indicating the sequence cluster (phylogroup). For each phylogroup, dashed lines represent the regression model estimate, and the gray shade on both sides of the dashed lines represents the 95% confidence interval. The correlation coefficient (*R*) and *P* values for each phylogroup are shown. Details of DefenseFinder results are presented in Table S5 and Figure S3.

We hypothesized that defense systems against phage DNA, mobile genetic elements, and other exogenous DNA are abundant but vary between the two phylogroups. Here, we examined the recombination output of Gubbins calculated from the core genome alignment. The recombined sequence length per genome was significantly different between phylogroup 2 and phylogroup 1 (*P* = 1.58 × 10^−49^, Welch’s *t*-test). We detected a total of 1,744 and 5,194 defense systems in phylogroup 2 (BAPS4) and phylogroup 1 (BAPS5), respectively (Table S5). Both phylogroups carry all five families of defense systems, with defenses related to nucleic acid degradation as the most common (Fig. S3). The two phylogroups also have the same set of most frequently detected defense systems, including restriction-modification, RloC, Gabija, CBASS, and CRISPR-Cas, identified using DefenseFinder (34). Comparing the genomes from phylogroup 1 (BAPS5) and phylogroup 2 (BAPS4), we observed a significant positive correlation between the total recombined sequence per genome with the number of defense systems per genome (Fig. 4; *R* = 0.262, *P* = 3.68 × 10^−11^ for phylogroup 1 and *R* = 0.056, *P* = 0.0042 for phylogroup 2).

### Network structure of shared accessory genes

Non-random gene co-occurrence has been postulated to arise as a result of a common evolutionary history, co-evolution of certain traits, linked response to selective pressures, or coordinated patterns of horizontal gene gain or gene loss (35, 36). We sought to examine gene co-occurrence in the accessory genomes of *P. aeruginosa*. Using Coinfinder (36), we obtained statistically significant co-occurrence of accessory genes in the two major phylogroups (Fig. 3a and b; Table S6; Fig. S2).

**Fig 5:**
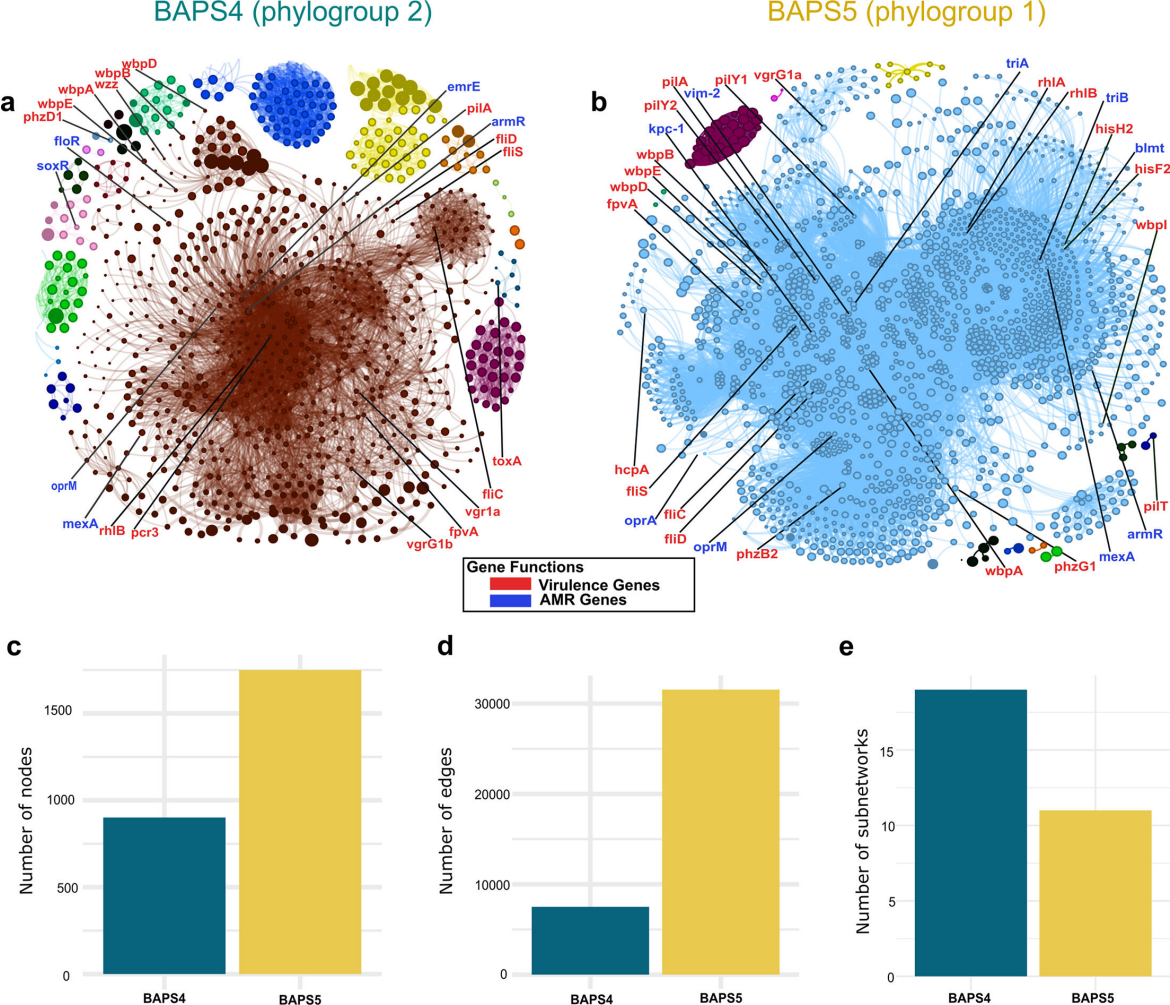
Co-occurrence of shared accessory genes in sequence clusters BAPS4 (phylogroup 2) and BAPS5 (phylogroup 1). Co-occurrence networks of sequence clusters (a) BAPS4 and (b) BAPS5. Nodes correspond to individual accessory genes, while edges linking them indicate significant gene associations. The subnetworks are represented by a distinct color. For visual clarity, only nodes representing antimicrobial resistance genes (blue nodes) and virulence genes (red nodes) are labeled. Comparison of the (c) number of nodes, (d) number of edges, and (e) number of subnetworks in the co-occurrence network of each sequence cluster. Details of Coinfinder results are found in Table S5.

In phylogroup 2 (BAPS4), we identified 901 genes in the co-occurrence network, which consisted of 7,508 co-occurring pairs and 19 subnetworks (Fig. 5a; Table S5). Within this network is a large subnetwork consisting of 648 nodes (colored brown in Fig. 5a), of which 14 are virulence genes and five are antimicrobial resistance (AMR) genes. The network contained co-occurring genes associated with florfenicol resistance (*floR*) and genes associated with multidrug efflux pumps (*armR, emrE, mexA, oprM, soxR*).

The co-occurrence network of phylogroup 1 (BAPS5) consisted of 1,750 genes and 31,549 co-occurring pairs, which were clustered into 11 subnetworks (Fig. 5b; Table S5). Within this network is a large subnetwork consisting of 1,690 nodes (colored light blue in Fig. 5b), of which 20 are virulence genes and nine are AMR genes. The network contained co-occurring genes associated with resistance to bleomycin (*blmt*)*,* penam (*kpc-1, vim-2*)*,* and triclosan (*triA, triB*)*,* as well as multidrug efflux pumps (*armR*, *mexA, oprA, oprM*).

In terms of virulence genes, phylogroup 2 (BAPS4) and phylogroup 1 (BAPS5) contained 16 and 21 co-occurring virulence genes, respectively. Functions of co-occurring virulence genes that were common between the two networks include those related to flagellin, siderophore pyoverdine biosynthesis, type III secretion system, type IV pili and twitching motility, rhamnolipid synthesis, type VI secretion system, and O-antigen lipopolysaccharide biosynthesis and regulation. In phylogroup 2 (BAPS4), we also detected co-occurring virulence genes related to phenazine biosynthesis and exotoxin. In phylogroup 1 (BAPS5), we also detected co-occurring virulence genes related to histidine auxotrophy.

In terms of the structure of the co-occurrence networks, phylogroup 1 contains higher number of nodes (Fig. 5c) and number of edges (Fig. 5d) in its network than phylogroup 2. However, phylogroup 1 had fewer subnetworks than phylogroup 2 (Fig. 5e). Overall, we found that the composition and structure of the networks of co-occurring accessory genes, including clinically relevant genes, varied between the two phylogroups.

## DISCUSSION

Our analysis of 840 globally distributed, ecologically diverse *P. aeruginosa* genomes highlights the tremendous variation in gene content within the species. Our main findings are as follows: (i) the two major phylogroups differ in the frequency and characteristics of homologous recombination in their core genomes, and (ii) different pools of AMR and virulence genes exist in the two phylogroups and display dissimilar patterns of co-occurrence. Altogether, our results indicate that the success of *P. aeruginosa* as an opportunistic pathogen lies in part on lineage-dependent patterns of DNA sharing in their core and accessory genomes. Similar findings on lineage-specific pan-genomes have also been reported in *Escherichia coli* (37), *Staphylococcus aureus* (38), and *Streptococcus agalactiae* (39). Our study further expands on these previous reports and contributes to the current discussion on the mechanisms and processes that drive the evolution of pan-genome content in prokaryotes (40–42).

Recombination causing replacement of homologous DNA segments generates polymorphic diversity and is a potent force in pathogen evolution (43). Our findings are consistent with previous reports of recombination in clinical *P. aeruginosa* (44–46). In our work, we show that recombination generates polymorphic diversity of core genes, but rates and impacts of recombination vary considerably between the two phylogroups. These differences may be attributed to barriers to inter-phylogroup recombination (47), although what these barriers are—ecological, mechanistic, adaptive (48)—in *P. aeruginosa* remain to be elucidated. Our observation of frequent recombination in both phylogroups compared to the moderate levels reported previously (32, 46) can be attributed to differences in the algorithms used to detect and estimate recombination. The previous study used the maximum likelihood-based clonal model implemented in ClonalFrame (49), which can underestimate recombination rates in data sets with intensive recombination (for in-depth comparison of recombination detection methods, please see reference 50). These results highlight the challenges of quantifying recombination across bacterial species, especially among very closely related strains, and thus a more nuanced interpretation of parameter estimates should be carefully considered. Regardless, frequently recombining core genes of various functions that are particularly relevant to host colonization and pathogenesis have experienced elevated frequencies of recombination, which likely contribute to the bacterium’s response to selection in the human host and as a strategy to evade host immunity.

Our findings that the two phylogroups exhibit distinct patterns of co-occurring accessory genes align with the concept of gene ecology, i*.*e., that genes occupy their own ecological niches, including the clonal background of the cell that carries them (51, 52). Thus, the maintenance of horizontally acquired accessory genes is determined by its interactions with other genes where it finds itself, reframing the pan-genome as a dynamic ecosystem (53). Such variation may reflect the importance of unique selective pressures (37), genetic drift (54), differential gene gain and loss over time (38, 55), epistatic relationships (56), and other evolutionary processes experienced by individual phylogroups that may be obscured when studying the pan-genome of the species as a whole. It may also reflect the existence of ecological micro-niches of the phylogroups and from which accessory genes can be sourced. The densely connected network (i*.*e., more nodes and edges) in sequence cluster BAPS5 (phylogroup 1) may be beneficial in conditions that require rapid adaptation, facilitated by gene products that are maintained, mobilized, and/or function together.

Pan-genome differences between the two phylogroups may also reflect variation in their immunity and defense systems that target phage DNA mobile genetic elements and other exogenous DNA (57), as we have shown and which has also been reported to vary among *P. aeruginosa* lineages (58, 59). Previous studies have shown that the distribution of defense systems is influenced by environmental variability (60) and is postulated to be a community resource, whereby individual strains benefit from the defense systems carried by close relatives (61). However, the unequal number of genomes between phylogroup 1 (BAPS5) and phylogroup 2 (BAPS4) in our study is also certainly an important consideration. Regardless, the impacts of recombination may likely be modulated by the notable diversity and abundance of defense systems in each phylogroup. The positive correlation we observed between the total length of recombined DNA and the number of defense systems was unexpected. However, we postulate that homologous recombination between strains of the same species is more permissive, but exogenous DNA derived from other species is likely to prompt an elevated number of defense systems. Future investigations should focus on the relationship between acquired DNA from other species and the defense systems of *P. aeruginosa*.

The global *P. aeruginosa* population consists of multiple epidemic and non-epidemic clones, with the former causing most clinical infections worldwide (16, 20, 62). The emergence and dissemination of multidrug-resistant epidemic clones are particularly troubling (62). Our analysis included the most notable epidemic clones: ST17, ST111, ST146, ST274, and ST395 in phylogroup 1 (BAPS5), and ST235 and ST253 in phylogroup 2 (BAPS4). Epidemic clones have emerged mainly through saltational evolution driven by horizontal gene transfer (16), thereby configuring clone-specific accessory genomes (16, 20, 63). Non-epidemic clones within each phylogroup can act as important reservoirs of AMR determinants from which epidemic clones can draw from, including polymorphic/allelic variants of AMR genes via homologous recombination and accessory AMR genes via gene gain. Non-epidemic clones can also act as transient intermediaries of less common AMR and virulence determinants, thereby preventing their loss from the population. Multidrug resistance can therefore emerge among different clonal backgrounds through the convergence of diverse AMR determinants. The same is true for virulence genes, although we found more similarities in virulence gene carriage between the two phylogroups. Future work on ST-specific genetic sharing will be particularly informative to precisely dissect the underlying basis and evolutionary drivers for the success of these epidemic clones.

We recognize the limitations of our study. First, the unequal representation of genomes from different geographical locations and ecological niches will certainly influence our estimation of the core and accessory genomes of the species and of individual phylogroups. We were not able to estimate the recombination and accessory genomes of phylogroups 3–5 due to their low number of genomes. This emphasizes the need to expand the surveillance of *P. aeruginosa* from a wider variety of environments and cryptic reservoirs (e*.*g., ventilation systems, sink drains, medical devices, food production systems) that may harbor rare lineages. Second, our inclusion of draft genome assemblies will certainly affect our pan-genome definition due to fragmented and incomplete sequences. While we ensured high-quality sequences are included in our analyses, additional long-read sequences to generate complete genomes will yield a comprehensive picture of the magnitude of the *P. aeruginosa* pan-genome, especially the identification of less common genes and singleton genes (i*.*e., genes held by a single genome). Long-read sequencing will also help distinguish accessory genes carried by chromosomes versus those genes carried by independently replicating plasmids, with the latter having more opportunities to be mobilized between strains. Lastly, we did not examine the genetic acquisitions (either via DNA replacement through homologous recombination or DNA addition through gene gain) from other *Pseudomonas* species or from other genera to *P. aeruginosa*. However, horizontal gene transfer between them is likely to occur because of shared ecology (i*.*e., co-existing in biofilms, the lung environment, the same hospital) and a common pool of mobile genetic elements.

In summary, our study reveals that distinct patterns of core genome recombination and co-occurrence of shared accessory genes differentiate the two major phylogroups of *P. aeruginosa*. Our findings provide important insights on the genetic determinants for the ecological versatility, virulence, and multidrug resistance features in the species. This knowledge will inform current efforts to control and manage diseases, including effective approaches to target specific *P. aeruginosa* phylogroups, clones, or lineages.

## MATERIALS AND METHODS

### Genome collection, sequence quality assessment, and annotation

We retrieved 1,000 publicly available genome sequence data (complete and draft genome assemblies) of *P. aeruginosa* compiled and previously published in reference (12). We used QUAST v.5.0.2 (64) and CheckM v.1.1.3 (65) to assess the quality of assembled genomes. Genomes with <90% completeness and >5% contamination were excluded. We also left out assemblies with >200 contigs and an N50 <40,000 bp to ensure that only high-quality genomes are included. All genomes were compared against each other using the program FastANI v.1.32 (66) to confirm that they belong to the same species, with the ≥95% average nucleotide identity (ANI) threshold (66). Genomes that did not meet the 95% ANI threshold were also excluded.

In all, a total of 840 genomes were used for all downstream analyses (Table S1). Genome completeness ranged from 98.71% to 100%, and genome contamination ranged from 0.08% to 2.96%, which were all within the genome quality standards recommended by CheckM (65). The number of contigs in this data set ranged from 1 to 199 (median = 87.5), and N50 ranged from 53.24 to 7,497.59 kbp (mean = 968.80 kbp). Genome sizes ranged from 5.46 to 7.55 Mbp. The resulting contigs were annotated using Prokka v.1.14.6 (67). The final data set consisted of genomes from 35 countries across Europe (*n* = 351 genomes), North America (*n* = 266), Asia (*n* = 106), and 43 genomes from unknown locations. The genomes were derived from four ecological niches: cystic fibrosis patients (*n* = 196), acute infections (*n* = 574), non-clinical environmental sources (*n* = 54), and animal sources (*n* = 16).

### Pan-genome analysis and phylogenetic tree reconstruction

We used Panaroo v.1.2.7 (68) to determine the core and accessory gene content of the entire population. We defined core genes as those present in ≥95% of the genomes (i*.*e., at least 798 genomes), whereas the accessory genes were genes present in ≥1% and <95% of the data set (i*.*e., less than 798 genomes). Panaroo was run in strict mode with the –remove-invalid-genes option to ignore annotations that do not conform to the expected gene structure (e*.*g., with a premature stop codon or gene of an invalid length). Gene sequences were aligned using MAFFT v.7.487 (69). Sequence alignments of 4,088 core genes were concatenated and from which SNPs were identified using SNP-site v.2.5.1 (70). A total of 1,119,257 SNPs were extracted. The aligned core SNPs were used to build a maximum likelihood phylogenetic tree using RAxML v.8.2.12 (71) with a generalized time reversible (GTR) (72) model of nucleotide substitution and gamma distribution of rate heterogeneity. We used the Bayesian hierarchical clustering algorithm fastBAPS v.1.0.6 (fast Bayesian Analysis of Population Structure) to partition the genomes into sequence clusters consisting of genetically similar individuals (73). We also built core SNP phylogenetic trees for individual sequence clusters using the same methods described above.

### *In silico* identification of ST, antimicrobial resistance, and virulence genes

STs were determined using the program mlst v.2.19.0 (https://github.com/tseemann/mlst), which extracts seven housekeeping genes (*acsA, aroE, guaA, mutL, nuoD, ppsA,* and *trpE* [30]) and compares allelic profiles against previously characterized STs in the *P. aeruginosa* PubMLST database (74). We used ABRicate v.1.0.1 (https://github.com/tseemann/abricate) to identify the presence of AMR and virulence genes (Table S7). We used the minimum thresholds of >80% for sequence coverage and >80% sequence identity for comparing query sequences from our data set with the curated sequences in the Comprehensive Antimicrobial Resistance Database (CARD) (75) and the Virulence Factor Database (VFDB) (76).

### Recombination detection

Using the core genome alignment as input and 1,000 bootstrapped replicates, we calculated the recombination rate using Mcorr with default parameters (31). Mcorr uses a coalescent-based model to measure the degree to which any two loci separated by *N* bp have correlated substitutions and estimates six evolutionary parameters: diversity (*d*), mutational divergence (θ), recombinational divergence (ϕ), relative rate of recombination to mutation (ϕ/θ), recombination coverage (*c*), and mean fragment size (*f*) of a recombination event (31).

We used Gubbins v.3.2.1 (33) to identify regions of recombination in the genome alignments. Gubbins uses a sliding window approach to identify regions in the genome containing elevated densities of SNPs. We ran Gubbins separately on each of the two major phylogroups. To identify the specific genes that were inferred to have recombined, we used snippy v.4.6.0 (https://github.com/tseemann/snippy) to align our genomes to the reference genome strain PAO1 (accession no. SAMN02603714). We used the default options in Gubbins: minimum number of SNPs to identify a recombination block = 3; minimum window size = 100 bp; and maximum window size = 10,000 bp. Recombination events were visualized using RCandy (77).

### Identification of accessory genome networks

To determine whether shared accessory genes co-occur more often than would be expected by chance, we used the program Coinfinder v.1.0.7 (36). Coinfinder detects genes that associate or dissociate with other genes using a Bonferroni-corrected binomial exact test statistic of the expected and observed rates of gene–gene association. Coincident genes were visualized as networks with Gephi v0.9.2 (78). Gene functions in the networks were determined from UniProt (79).

### Detection of anti-phage defense systems

We carried out an *in silico* detection of defense systems against phage DNA, mobile genetic elements, and exogenous DNA using DefenseFinder v. 2.0.0 (34) and implementing the macromolecular systems detector program MacSyFinder v.2 (80). DefenseFinder uses homology search and hidden Markov models to build a profile analysis of specific genes and gene architecture associated with known antiphage systems.

### Statistical analysis

We carried out all statistical analysis using the ggstatsplot v.0.12.1 (81) and visualization using the ggplot2 v.3.4.4 (82) package in R v.4.3.1 (83). We used Welch’s *t*-test to compare the Mcorr parameters between samples. Pearson correlation test and linear regression analysis were used to determine the relationship between recombination and bacterial anti-phage defense systems. Results were considered significant when *P* < 0.05. Unless otherwise noted, default parameters were used for all programs.

## Data Availability

The data set supporting the conclusions of this article is included within the article and its supplemental files. Genome sequence data of *P. aeruginosa* are publicly available in the Short Read Archive database of the National Center for Biotechnology Information (NCBI) and published in reference 12. BioSample accession numbers for each genome are listed in Table S1.

## SUPPLEMENTAL MATERIAL

Supplemental figures msystems.00301-25-s0001.pdf Figure S1–S3.

Supplemental tables msystems.00301-25-s0002.xlsx Tables S1–S7.

ASM does not own the copyrights to Supplemental Material that may be linked to, or accessed through, an article. The authors have granted ASM a non-exclusive, world-wide license to publish the Supplemental Material files. Please contact the corresponding author directly for reuse.
