# Supplementary material for: Heterogeneity in recombination rates and accessory gene co-occurrence distinguish Pseudomonas aeruginosa phylogroups
Source: mSystems. 2025 Apr 30;10(5):e00301-25. doi: 10.1128/msystems.00301-25 (PMC12090758; doi:10.1128/msystems.00301-25)
Supplement: Supplemental figures — Figure S1–S3. [file msystems.00301-25-s0001.pdf]

Supplementary figures

Heterogeneity in recombination rates and accessory gene co-occurrence distinguish *Pseudomonas aeruginosa* phylogroups

Samara T. Choudhury, Kathryn R. Piper, Manuela Montoya-Giraldo, Odion O. Ikhimiukor, Jeremy R. Dettman, Rees Kassen, Cheryl P. Andam

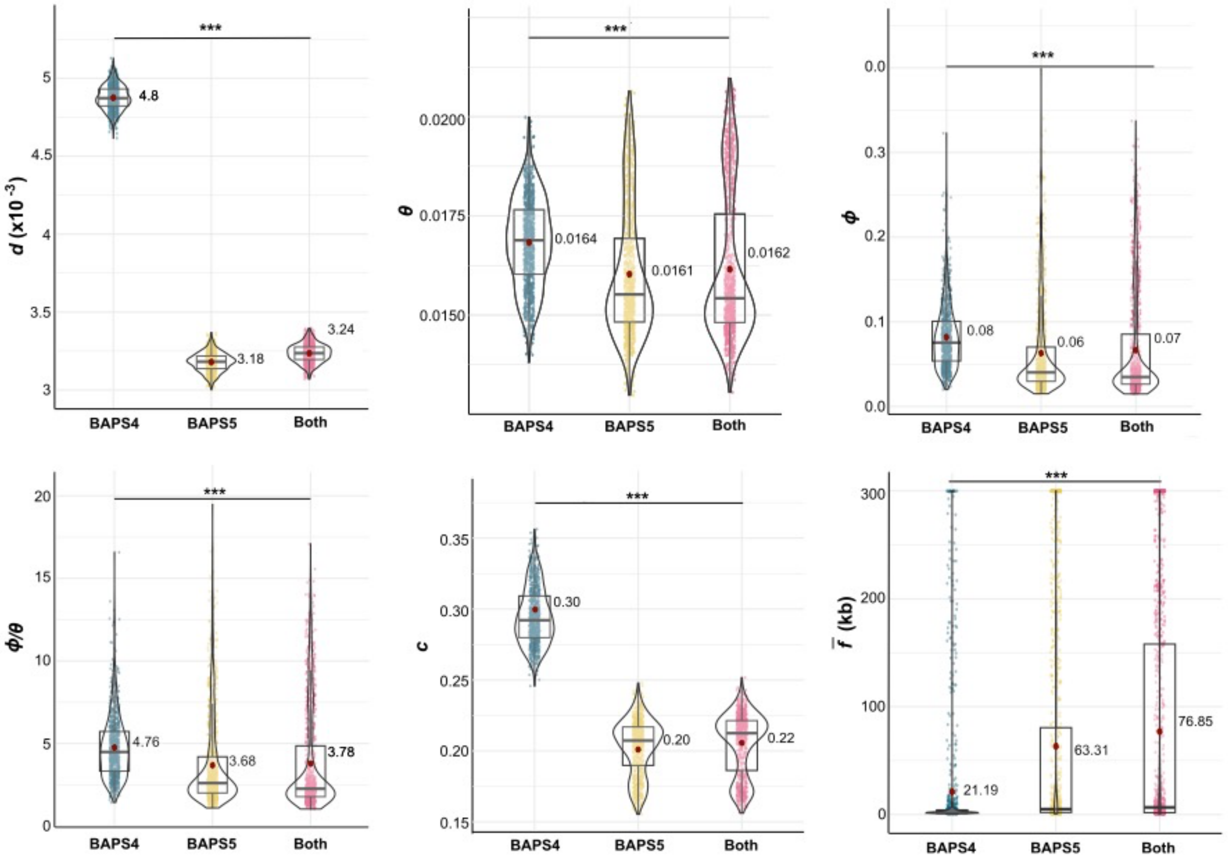

Supplementary Figure S1. Comparison of recombination parameters in phylogroup 1 (BAPS5) only, phylogroup 2 (BAPS4) only, and combined BAPS5+BAPS4. Parameters were calculated using Mcorr.

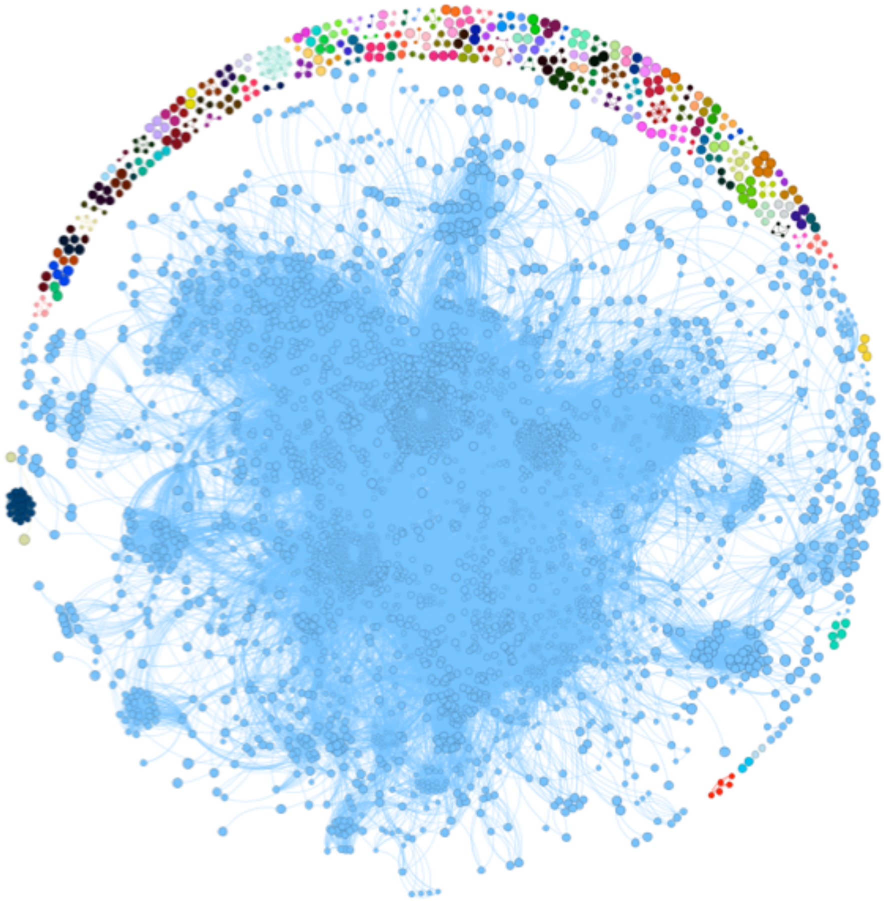

Supplementary Figure S2. Co-occurrence network of shared accessory genes in the combined dataset of BAPS4 (phylogroup 2) and (b) BAPS5 (phylogroup 1).

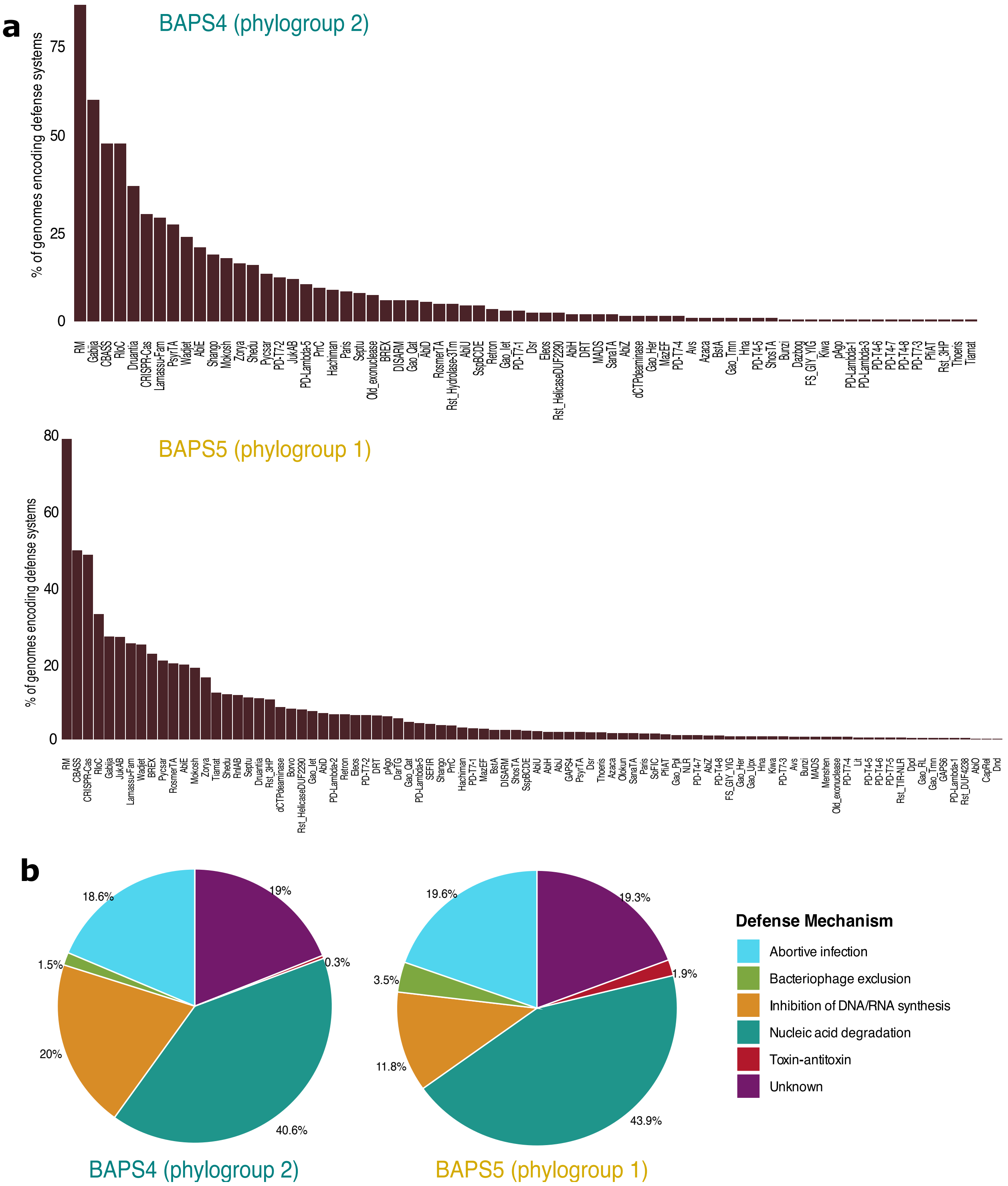

Supplementary Figure S3. Defense systems detected in *P. aeruginosa* genomes identified using DefenseFinder (reference 34). Frequency and types of defense systems in sequence clusters (a) BAPS4 (phylogroup 2) and (b) BAPS5 (phylogroup 1). A genome may contain more than one defense system. Classification of the defense mechanisms and their proportion in sequence clusters (c) BAPS4 (phylogroup 2) and (d) BAPS5 (phylogroup 1).
